# Low-intensity muscle contraction exercise reduces pain sensitivity by modulating peripheral pathology and spinal sensitization in end-stage knee osteoarthritis rats

**DOI:** 10.3389/fpain.2025.1644177

**Published:** 2025-09-29

**Authors:** Satoko Motokawa, Junya Sakamoto, Ryo Sasaki, Yuki Nishi, Yuichiro Honda, Ayumi Takahashi, Minoru Okita

**Affiliations:** ^1^ Department of Rehabilitation, Sakurajyuji Fukuoka Hospital, Fukuoka, Japan; ^2^ Department of Physical Therapy Science, Nagasaki University Graduate School of Biomedical Sciences, Nagasaki, Japan; ^3^ Institute of Biomedical Sciences (Health Sciences), Nagasaki University, Nagasaki, Japan; ^4^ Japan Society for the Promotion of Science, Tokyo, Japan

**Keywords:** osteoarthritis, pain, peripheral pathology, central sensitization, low-intensity muscle contraction exercise

## Abstract

**Introduction:**

This study evaluated the effects of low-intensity muscle contraction exercise on pain sensitivity, peripheral pathology, and central sensitization in the spinal dorsal horn in a rat model of end-stage knee osteoarthritis (OA) induced by monosodium iodoacetate (MIA).

**Methods:**

Sixty-two male Wistar rats were assigned to three groups: OA, Exercise, and Sham. The Exercise group underwent quadriceps muscle contractions induced by electrical stimulation (50 Hz, 250 µs, 2–3 mA) with a duty cycle of 1:2 (2 s On, 4 s Off) for 20 min daily, five days per week, from day 29 to day 56 post-MIA administration. Pain sensitivity was assessed by measuring knee joint pressure pain thresholds (PPT) and paw withdrawal thresholds using von Frey filaments. Histological and immunohistochemical analyses of the knee joint and spinal cord included toluidine blue staining, tartrate-resistant acid phosphatase staining, and markers for CD68, CD11c, CD206, osteoclasts, nerve growth factor (NGF), calcitonin gene-related peptide (CGRP), and phosphorylated NR1 (pNR1).

**Results:**

Knee joint PPTs were significantly higher in the Exercise group after day 35, accompanied by reductions in CD68-, CD11c-positive cells, NGF-positive cells, CGRP-positive fibers, osteoclasts, and pNR1-positive cells, as well as an increase in CD206-positive cells, compared to the OA group.

**Discussion:**

Despite no significant changes in cartilage or subchondral bone degeneration, these findings suggest that low-intensity muscle contraction exercise alleviates pain sensitivity by modulating peripheral pathology and central sensitization. This study highlights the potential of therapeutic exercise as a strategy for OA pain management.

## Introduction

Several guidelines recommend exercise as a core treatment for knee osteoarthritis (OA) (1–3). A systematic review of randomized controlled trials (RCTs) included in these systematic reviews and meta-analyses revealed that most studies have focused on patients with advanced knee OA, classified as grade I–III according to the Kellgren/Lawrence (K/L) classification. In addition, basic research using animal models of knee OA has confirmed the pain-relieving effects of exercise therapy across different stages of OA (4, 5). Thus, exercise therapy can provide pain relief for patients with advanced knee OA. However, few studies have evaluated the effects of exercise therapy on pain reduction in patients with end-stage knee OA (grade IV K/L classification), and their results remain inconsistent (6–9). For example, Skoffer et al. reported significant pain improvement in end-stage knee OA patients who underwent 4 weeks of quadriceps muscle contraction exercises (6), and another report describes pain reduction after 8 weeks of similar exercises (7). In contrast, other studies reported no pain reduction after 6 weeks (8) or 12 weeks (9) of quadriceps exercises. These inconsistencies may be due to differences in the intervention setting, frequency and duration. We speculated that this lack of clarity could be resolved by investigating the precise pain-reducing effects of exercise in animal experiments.

The pathogenesis of pain in end-stage knee OA is complex and involves both peripheral tissues of the knee joint and pain sensitization. Synovitis is strongly correlated with pain severity (10), and macrophage infiltration into the synovium is a hallmark of OA (11). M1 macrophages produce proinflammatory cytokines such as IL-1β and TNF-α that exacerbate synovitis, while M2 macrophages secrete anti-inflammatory mediators including IL-10 that suppress inflammation (12). Clinical studies have reported an imbalance of increased M1 and decreased M2 macrophages in the synovium of OA patients with severe pain compared with healthy individuals, further supporting their involvement in OA pain (13). Thus, evaluating macrophage polarization markers (CD68 for total macrophages, CD11c for M1, and CD206 for M2) is critical for understanding exercise-induced changes in synovial inflammation.

In addition, subchondral bone changes and osteoclast activation contribute to knee OA pain. Activated osteoclast produces nerve growth factor (NGF), which act as a pain mediator and stimulates primary sensory neurons in the subchondral bone, resulting in pain originating from the subchondral bone (14–16). These peripheral changes cause central sensitization, as reflected by increased expression of phosphorylated NR1 (pNR1) in spinal dorsal horn neurons (17).

The analgesic effects of exercise in end-stage knee OA remain unclear, and the biological mechanisms underlying these effects have not been elucidated. We hypothesized that low-intensity muscle contraction exercise would alleviate pain in end-stage knee OA, and that this effect would be associated, at least in part, with modulation of synovial inflammation, knee OA-related pathological changes in subchondral bone, and central sensitization in the spinal dorsal horn. To address this hypothesis, the present study had two objectives. First, we aimed to clarify whether low-intensity muscle contraction exercise induced by electrical stimulation exerts analgesic effects in a rat model of end-stage knee OA under controlled experimental conditions. Second, if such effects were observed, we sought to investigate the potential pathophysiological mechanisms by examining synovial macrophages (CD68: all macrophages, CD11c: M1 type, CD206: M2 type), osteoclast activity, NGF expression, CGRP-positive sensory neurons in the subchondral bone, and phosphorylated NR1 (pNR1) in the spinal dorsal horn as a marker of central sensitization.

## Material and methods

### Animals

Seven-week-old male SPF Wistar rats (CLEA Japan, Tokyo) were housed at the Nagasaki University Center for Frontier Life Sciences. The rats were maintained in 30 × 40 × 20-cm cages (two rats per cage) under a 12-h light-dark cycle (lights on from 7:00 to 19:00) at an ambient temperature of 25°C. Food and water were provided freely. A total of 62 rats were included in the study.

The rats were randomly assigned to one of three groups: (i) The OA group, in which right-knee osteoarthritis was induced as described below; the rats then lived under the standard housing conditions for 56 days. (ii) The Ex group, in which OA was induced and quadriceps muscle contraction exercises were initiated 28 days later, continuing for another 28 days; and (iii) the sham rats, which received saline injections as a sham treatment and were maintained under the standard housing conditions for 56 days.

The primary endpoint of this study was pain sensitivity, while histological changes in the synovium were evaluated as a secondary endpoint. Because comparable data for pain sensitivity in this OA model were not available, the sample size was calculated using immnohistological data from our group's previous study (18). With an alpha error of 0.05 and a statistical power of 0.8, the required sample size was nine animals per group. To account for potential dropouts, we set the number at 10 rat per group. Accordingly, we estimated a total of 20 animals would be needed, 10 in each group for the macrophage-count analysis and 10 in each group for other histological analysis described below. Finally, each group was assigned 20 rats. Randomization was performed using serial numbers generated in Excel, but potential confounders such as treatment order and measurement sequence were not controlled.

All protocols for the animal procedures were reviewed and approved by the Ethics Review Committee for Animal Experimentation at Nagasaki University (No. 1808091472–9). All experimental procedures were performed in accord with the Guidelines for the Proper Conduct of Animal Experiments issued by the Science Council of Japan. The rats were eventually sacrificed under anesthesia with a mixture of medetomidine (0.375 mg/kg), midazolam (2.0 mg/kg), and butorphanol (2.5 mg/kg) to prevent/ease suffering. We did not have the strategy used to minimize potential confounders such as the order of treatments or animal/cage location.

### The OA model: intra-articular injection of mono-iodoacetate

Monosodium iodoacetate (MIA) induces joint pathology by inhibiting glycolysis, leading to chondrocyte death and secondary OA. We have observed that a 2-mg dose of MIA causes a long-term decrease in the tenderness threshold of the rat knee joint (18). For the present experiment, rats were first anesthetized with a mixture of medetomidine (0.375 mg/kg), midazolam (2.0 mg/kg), and butorphanol (2.5 mg/kg). Next, 50 µl of sterile saline containing 2 mg of MIA (Sigma-Aldrich, St. Louis, MO, USA) was injected via a 30-gauge needle into the right knee joint via the patellar ligament. The sham group received 50 µl of sterile saline as a control. This procedure was performed by a single investigator (JS) and concealed from other researchers throughout the study. No animals showed signs of infection or significant distress after the injection. Studies using this method have reported near-complete cartilage loss 28 days post-MIA injection, corresponding to end-stage OA (19). Humane endpoints were established in accordance with ethical guidelines. Specifically, if signs of injection-related infection were observed, the animal was considered to have reached a humane endpoint. In such cases, euthanasia was performed by carbon dioxide inhalation to minimize suffering.

### Low-intensity muscle contraction exercise

Quadriceps muscle contraction exercises began on day 29 post-MIA injection, corresponding to the end-stage OA model. The protocol was as described (20). Briefly, muscle contraction was induced with the use of a commercial electrical stimulation device (Trio 300; Ito Physiotherapy and Rehabilitation, Tokyo). Electrode pads were placed on the medial and lateral aspects of the rat's right thigh. Stimulation was applied at a frequency of 50 Hz, pulse width of 250 µs, and intensity of 2–3 mA, with a duty cycle of 1:2 (2 s on, 4 s off) for 20 min/day, 5 days/week for 28 days.

### Pain behavioral assessment

On before and after administration of MIA or saline, on days 4 and 7, and every 7 days thereafter until day 56, 10 rats in each of the groups were subjected to a pain behavioral evaluation that measured the pressure pain threshold (PPT) of the right knee joint and the paw withdrawal response (PWR) of the hind paw to mechanical stimulation, as described (18, 21). Behavioral assessments were conducted by experimenters who were blinded to group assignments. Animals were identified solely by coded numbers, and the assignment key was retained by a separate researcher not involved in testing. Because the codes were distributed across groups, assessments were performed in numerical order, which effectively randomized the testing sequence between groups. To minimize variability, animals were acclimated to the testing environment and apparatus for 10 min per day over 7 consecutive days before baseline measurement. Furthermore, to prevent anticipatory responses during repeated measurements, the same animal was not tested consecutively; instead, evaluations were alternated with those of other animals. These measures were implemented to minimize bias, although we acknowledge that group assignment could still have been inferred if spontaneous pain-related behaviors differed markedly between groups.

The PPT of the right knee joint was measured with a Randall-Selitto apparatus (Ugo Basile, Varese, Italy). The transducer probe (9-mm dia.) applied increasing pressure (48 g/s) to the lateral side of the right knee joint. The pain threshold was defined as the force required to elicit hind-limb flexion or vocalization.

The PWR was assessed with the use of a von Frey sensory apparatus (IITC Life Science, Woodland Hills, CA, USA). The rat was placed in an acrylic cage with a mesh floor, and mechanical stimulation was applied to the plantar region of the right foot via a conical rigid plastic tip. The threshold was recorded as the pressure (g) causing the paw withdrawal reflex. The PPT and PWR were each measured five times per rat, and the average of the three middle values was used. To minimize potential confounders, no specific controls were used.

### Tissue sampling and preparation

At the end of the experimental period, the rats were anesthetized and perfused with saline followed by 4% paraformaldehyde (PFA) in phosphate-buffered saline (PBS, pH 7.4) for tissue fixation. The right knee joint and the spinal cord (L2/3, L4/5) were extracted. The knee joints were post-fixed in 4% PFA for 24 h and then demineralized in Morse's solution; The knee joint of 15 animals in each group was embedded in paraffin. The remaining knee joint and spinal cords were immersed in a 10% sucrose solution followed immersing in a 30% sucrose solution, and then embedded in optimal cutting temperature (OCT) compound before being stored at −80°C.

### Histological analysis of the knee joints

The frontal sections (5-µm thick) from paraffin-embedded knee joints of 10 rats per group were stained with toluidine blue stain. The cartilage and subchondral bone lesions were scored using the Osteoarthritis Research Society International (OARSI) grading system (22) under a light microscope (ECLIPSE 50i, Nikon, Tokyo). Cartilage degeneration was scored from 0 (normal) to 5 (severe) points in three zones of the tibial plateau, and the scores were summed. Subchondral bone damage was similarly graded from 0 to 5. The most severe lesion of the tibial plateau in each section was scored. The analyses were performed by an evaluator who was unaware of the treatment groups.

### Histochemical analysis

#### Analysis of osteoclasts (TRAP staining)

Five-µm-thick frontal sections from paraffin-embedded knee joints of 10 rats per group were subjected to tartrate-resistant acid phosphatase (TRAP) staining. Using an all-in-one microscope (BZ-X800, Keyence, Osaka, Japan), TRAP staining images were captured at a magnification of 200×. Osteoclasts were manually counted in each image, with positive cells defined as TRAP-positive cells showing a clear signal and containing multiple nuclei. The length of the bone marrow cavity margin in the subchondral bone was measured with the segmented line tool in ImageJ software (ver. 1.51, NIH, Bethesda, MD, USA), and “Measure” function was used to calculate the total traced length. The lengths of all traced margins within a section were summed, and osteoclast number were normalized to this value (cells/mm). Two sections were analyzed for each animal.

### Immunohistochemical analysis

#### Analysis of synovial macrophages

We employed CD68 as a pan-macrophage marker, CD11c as an M1-like marker, and CD206 as an M2-like marker, based on prior studies demonstrating their use for distinguishing macrophage phenotypes (23). Two frontal 5-µm-thick sections from paraffin-embedded knee joints of 10 rats per group were subjected to an antigen retrieval step by incubation in 0.01 M citrate buffer (pH 6.0) followed by a 4-min incubation at 90°C and then a 30-min incubation with 0.3% H_2_O_2_ in methanol. The sections were blocked with 1% bovine serum albumin (BSA) containing 1% normal horse serum for 60 min. Next, the sections were incubated with a mouse anti-CD68 monoclonal antibody (Cat No. MCA341R, 1:200; Bio-Rad, Hercules, CA), rabbit anti-ITGAX/CD11c polyclonal antibody (Cat No. LS-B15988, 1:2,000; LifeSpan Biosciences, Seattle, WA), and rabbit anti-mannose receptor polyclonal antibody (Cat No. ab64693, 1:3,000; Abcam, Cambridge, MA) for 30 min, followed by incubation with biotinylated horse anti-mouse IgG (BA-2000, 1:2,000; Vector Laboratories, Burlingame, CA) or biotinylated goat anti-rabbit IgG (H + L) (BA-1000; 1:3,000; Vector Laboratories). Each section was stained using an avidin-biotin complex method (Vectastain Elite ABC kit; Vector Laboratories) and then visualized with a metal-enhanced DAB substrate kit (Thermo Fisher Scientific, Waltham, MA). Each section was stained with methyl green. The medial and lateral synovium were photographed at 400×. For each image, the region of interest was defined as a 100-µm zone extending from the synovial lining toward the fibrous layer. Within this area, positive cells were manually counted, with positivity defined as cells showing a clear chromogenic signal. The area of the region of interest was measured using polygon tool in ImageJ software, and macrophage density was calculated as the number of positive cells per mm^2^. As the antibodies used in this study recognize cytoplasmic glycoproteins (CD68) or transmembrane glycoproteins (CD11c and CD206), we defined a positive signal as immunoreactivity that was observed encircling the entire nucleus of a cell. Two sections were analyzed for each animal. Each immunohistochemical staining was performed on separate adjacent sections. As a result, the numbers of positive cells for each marker are not intended to represent strict totals or to be directly comparable across markers, since one-to-one correspondence of individual cells cannot be ensured.

#### Analysis of NGF in subchondral bone

NGF-positive cells in the subchondral bone were similarly analyzed using the knee joint of five rats per group. Sagittal sections were incubated in 0.01 M citrate buffer (pH 6.0) at 80°C for 10 min followed by 0.6% H_2_O_2_/methanol for 40 min. Anti-β-NGF antibody (clone 1F18, 1:1,000; ZooMAb® rabbit monoclonal antibody, Millipore Sigma, St. Louis, MO) was used, and the other treatments were as described above. The measurement of the number of positive cells in subchondral bone was performed in the same manner as that used for the osteoclasts. The individual performing the analysis was blind to the rats' groups.

### Immunofluorescence analysis

#### Analysis of CGRP-positive fibers in subchondral bone

We conducted an immunofluorescence analysis of calcitonin gene-related peptide (CGRP)-positive fibers in the subchondral bone by using 20 µm-thick sagittal sections from frozen samples of five rats per group. After a 5-min fixation with 70% alcohol, the sections were blocked in 5% BSA/PBS for 2 h and then reacted with the primary antibody (anti-calcitonin gene-related peptide, 1:2,000, Immunostar, Hudson, WI) for 24 h at room temperature, followed by the secondary antibody (goat anti-rabbit IgG conjugated to Texas Red®, 1:2,000, Vector Laboratories) for 60 min at room temperature. The nuclei were stained with DAPI and sealed using Vectashield® mounting medium with DAPI (Vector Laboratories). Fluorescent images were captured at 200× magnification. In each image, CGRP-positive fibers were manually counted, with positive fibers defined as those exhibiting a clear fluorescent signal. Measurements of the bone marrow cavity were performed using images captured at 20× magnification, following the same procedure as described for osteoclast measurements. The density of CGRP-positive fibers was expressed as the number of positive fibers per mm^2^ of bone marrow cavity. Two sections were analyzed per animal.

#### Analysis of pNR1 in the spinal cord dorsal horn

Serial 10-µm-thick frozen transverse sections from 10 rats per group were prepared. One of the sections was used for fluorescence immunostaining, and another section was used for hematoxylin and eosin (H&E) staining. Blocking was first performed with 1% BSA. Then, anti-phosphor NR1 antibody (1:1,000, Millipore Sigma) was used as the primary antibody and reacted overnight at 4°C. Goat anti-rabbit IgG conjugated to Texas Red® (1:1,000, Vector Laboratories) was used as the secondary antibody, and the section was reacted at room temperature for 60 min. Nuclear counterstaining was performed as described above. Fluorescent images were captured at 200× magnification. In each dorsal horn, pNR1-positive cells were manually counted, with positive cells defined as those exhibiting a clear fluorescent signal and containing distinct nucleus. H&E-staining sections were obtained at 40× magnification. The area of the dorsal horn was measured manually using polygon tool in ImageJ software, and calculated with the “Measure” function. The density of pNR1-positive cells was expressed as the number of positive cells per mm^2^ of dorsal horn. Two sections were analyzed for each rat.

### Statistical analysis

All statistical analyses were performed using SPSS version 25.0 (IBM Corp., Armonk, NY, USA) or EZR (version 4.0.2), as appropriate. Data are presented as mean ± standard deviation, with the exception of the cartilage and subchondral bone scores. For behavioral outcomes (PPT and PWR), a linear mixed-effects model with group (Sham, OA, Ex), time (days after MIA injection), and their interaction as fixed effects, and subject ID as a random effect. *Post hoc* comparisons were conducted with Bonferroni correction to adjust for multiple testing. Data distribution was assessed with the Shapiro–Wilk test and by visual inspection of Q–Q plots. If the data satisfied the assumptions of normality (Shapiro–Wilk test, *p* > 0.05), parametric tests were applied. A one-way ANOVA was used to evaluate the cell counts with Bonferroni's method for *post-hoc* comparisons. For comparisons of cartilage and subchondral bone degeneration scores, data were analyzed using the Kruskal–Wallis test, followed by pairwise *post hoc* comparisons with the Steel–Dwass method. Statistical significance was set at *p* < 0.05.

## Results

### Between-group differences in the PPT and PWR

**Figure 1:**
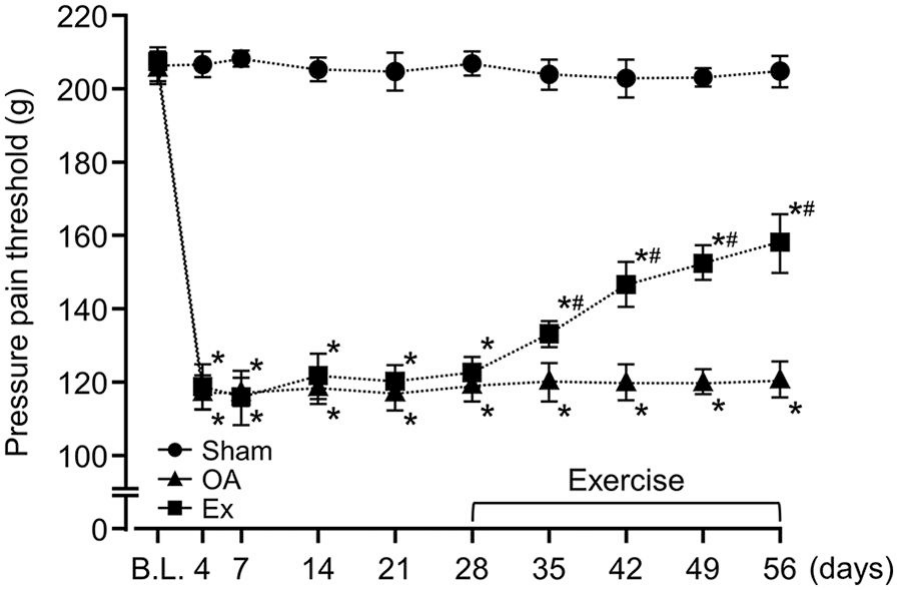
Changes in the pressure pain threshold (PPT) of the right knee joint over the 56-day study period. *vs. the Sham group, #vs. the OA group, *p* < 0.05.

**Figure 2:**
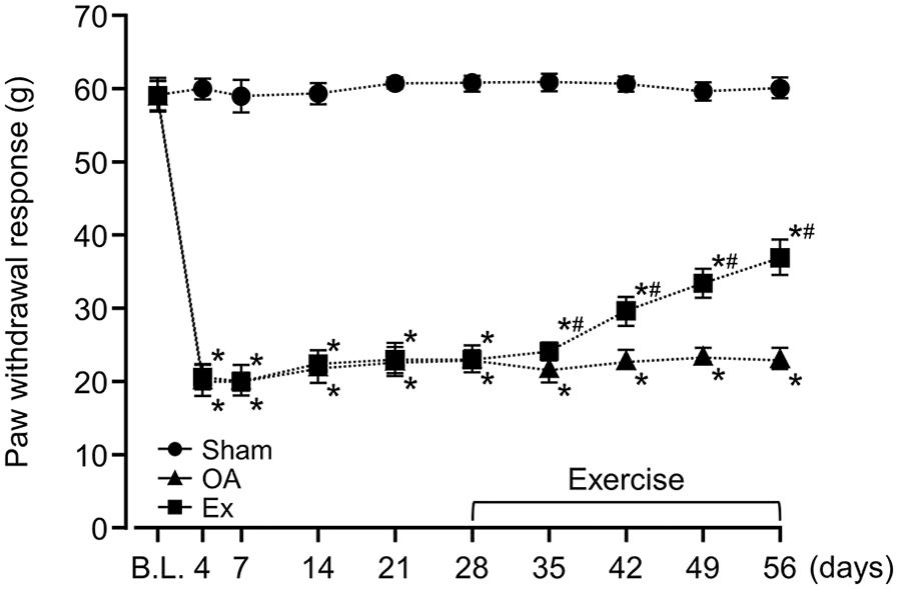
Changes in the paw withdrawal threshold (PWR) of the right knee joint over the 56-day study period. *vs. the Sham group, #vs. the OA group, *p* < 0.05.

After the administration of MIA, the rats in both the OA group and the Exercise group had significantly lower PPT and PWR values than the sham group, a difference that persisted throughout the experimental period (Figures 1, 2 and Supplementary Table 1). For example, on day 7, PPT was 208.7 ± 2.3 g in Sham vs. 121.1 ± 5.6 g in OA (*p* < 0.001) and 116.1 ± 7.8 g in Ex (*p* < 0.001), with similar patterns for PWR. On day 35 post-MIA injection (day 7 of the exercise intervention), the Exercise group showed significantly higher PPT (OA; 120.9 ± 6.9 g, Ex; 133.4 ± 3.7 g, *p* < 0.001) and PWR (OA; 21.8 ± 2.1 g, Ex; 24.0 ± 1.3 g, *p* < 0.001) than the OA group. This intergroup difference was maintained until the end of the experimental period (Figures 1, 2 and Supplementary Table 1).

### Histological evaluation of the knee joints

**Figure 3:**
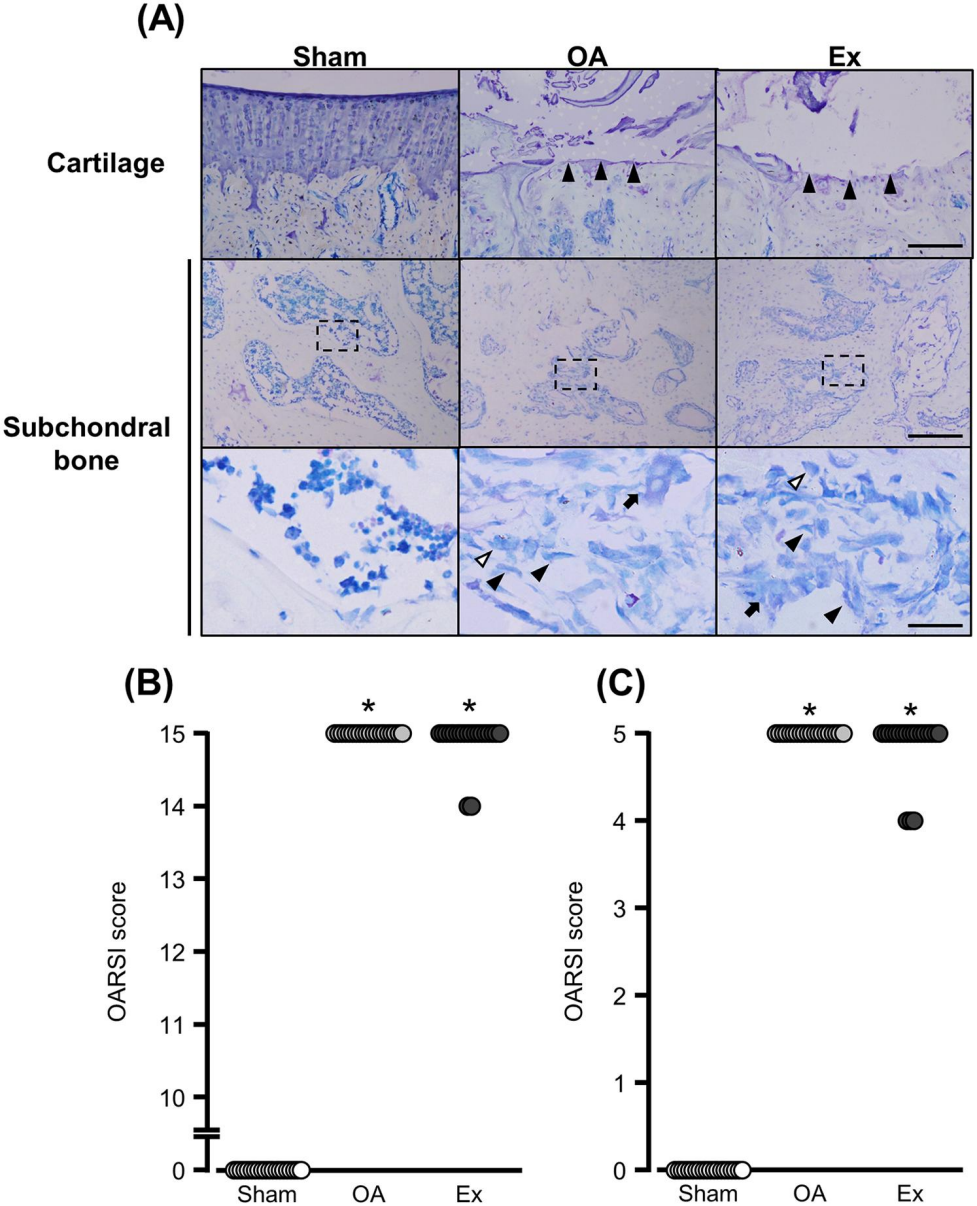
Histological findings with toluidine blue staining and degeneration scores in cartilage subchondral bone. **(A)** Representative histological findings, Black arrowhead in the bottommost image: fibroblast-like cell. White arrowhead in the bottommost image: macrophage-like cell. Arrow in the bottommost image: osteoclast-like cell. Bar (cartilage): 150 µm. Bars (subchondral bone): 150 µm (*upper*) and 30 µm (*lower*). **(B)** Cartilage degeneration scores. **(C)** Subchondral bone degeneration scores. *vs. the Sham group, *p* < 0.05.

The OA and Ex rats exhibited substantial articular cartilage loss (Figure 3A upper panels) and spindle-shaped cells that resembled fibroblasts had accumulated in the bone marrow cavity directly beneath the cartilage (Figure 3A middle/lower panels). The median articular cartilage degeneration scores were 0 [0–0] for the Sham group, 15 [15–15] for the OA group, and 15 [14–15] for the Ex group. Both the OA and Ex groups had significantly higher scores than the Sham group (Sham vs. OA, *p* < 0.001; Sham vs. Ex, *p* < 0.001), whereas no significant difference was observed between the OA and Ex groups (*p* = 0.324) (Figure 3B). Similarly, the median subchondral bone degeneration scores were 0 [0–0] for the Sham group, 5 [5–5] for the OA group, and 5 [4–5] for the Ex group. Both the OA and Ex groups had significantly higher scores than the Sham group (Sham vs. OA, *p* < 0.001; Sham vs. Ex, *p* < 0.001), while no significant difference was found between the OA and Ex groups (*p* = 0.177, Figure 3C).

### Results of the immunohistochemical analysis of macrophage markers

**Figure 4:**
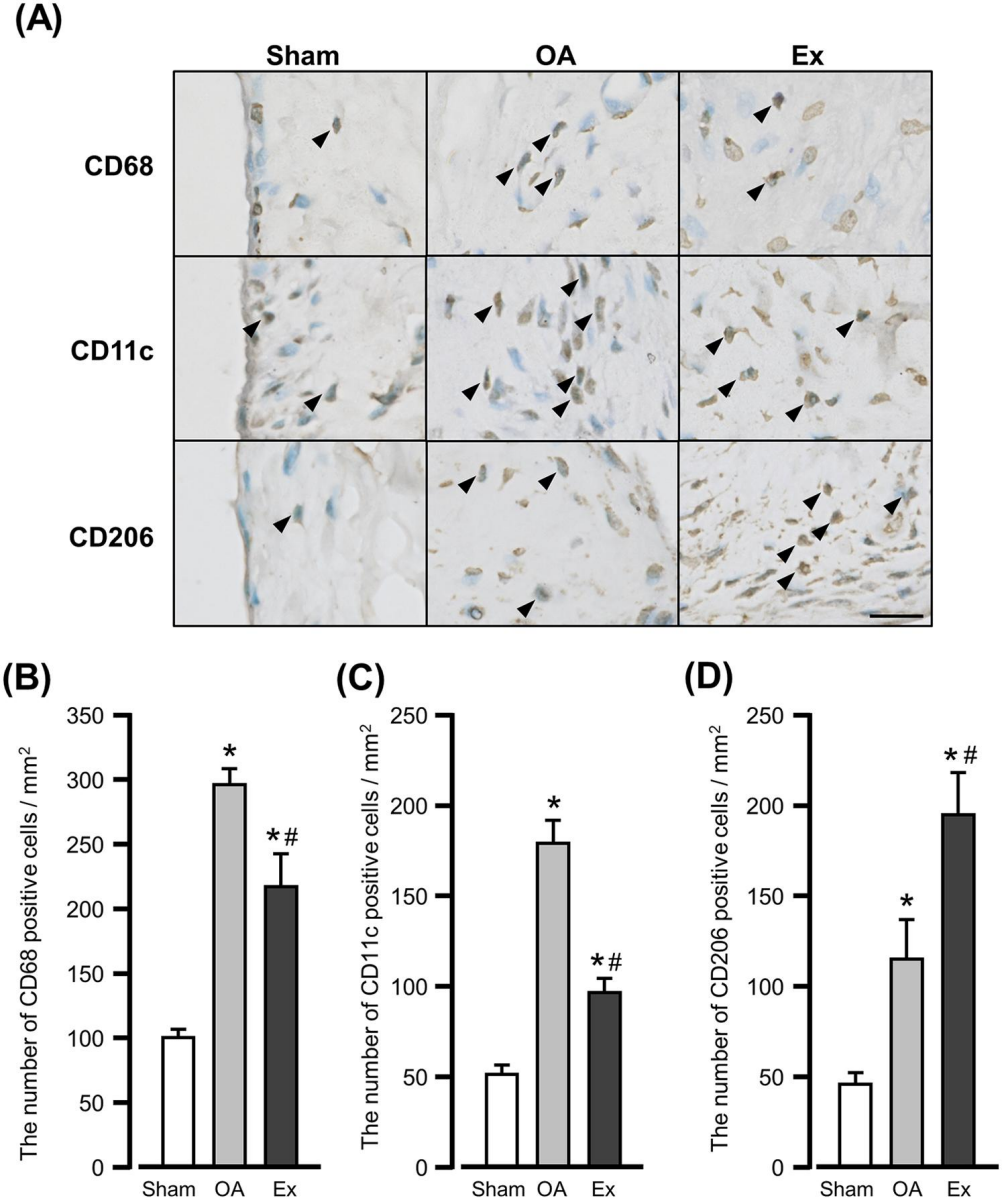
Immunohistochemical findings and quantification of immunoreactive cells for macrophage markers in the synovium. **(A)** Representative immunohistochemical findings show the CD68-, CD11c-, and CD206-positive cells in the synovium of the right knee joint at 56 days after MIA injection. *Arrowhead:* positive cells. Bar: 10 µm. **(B)** The number of CD68-positive cells. **(C)** The number of CD11c-positive cells. **(D)** The number of CD206-positive cells. *vs. the Sham group, #vs. the OA group, *p* < 0.05.

In the sham group, CD68-, CD11c-, and CD206-positive cells were observed in the superficial layers of the synovium (Figure 4A left panels). In the OA group, the positive cells were widely distributed from the inner to the subintimal layers of the synovium (Figure 4A center panels). The Ex group also showed positive cells in these layers but less for CD68- and CD11c- and more for CD206-positive cells compared to the OA group (Figure 4A right panels).

The mean number of CD68-positive cells was 101.3 ± 4.7 cells/mm^2^ in the Sham group, 295.6 ± 11.8 cells/mm^2^ in the OA group, and 217.2 ± 25.7 cells/mm^2^ in the Ex group. Both the OA and Ex groups had significantly higher values than the Sham group (Sham vs. OA, *p* < 0.001; Sham vs. Ex, *p* < 0.001), while the Ex group had significantly lower values than the OA group (*p* < 0.001) (Figure 4B).

For CD11c-positive cells, the mean values were 50.1 ± 5.4 cells/mm^2^ in the Sham group, 178.6 ± 12.8 cells/mm^2^ in the OA group, and 97.3 ± 6.2 cells/mm^2^ in the Ex group. Both the OA and Ex groups showed significantly higher values compared with the Sham group (*p* < 0.001 for both), and the Ex group had significantly lower values than the OA group (*p* < 0.001, Figure 4C).

Concerning CD206-positive cells, the mean values were 44.5 ± 6.5 cells/mm^2^ in the Sham group, 114.4 ± 22.8 cells/mm^2^ in the OA group, and 194.0 ± 24.1 cells/mm^2^ in the Ex group. Both the OA and Ex groups had significantly higher values than the Sham group (*p* < 0.001 for both), and the Ex group had significantly higher values than the OA group (*p* < 0.001, Figure 4D).

### Osteoclast, NGF, and CGRP values in subchondral bone

**Figure 5:**
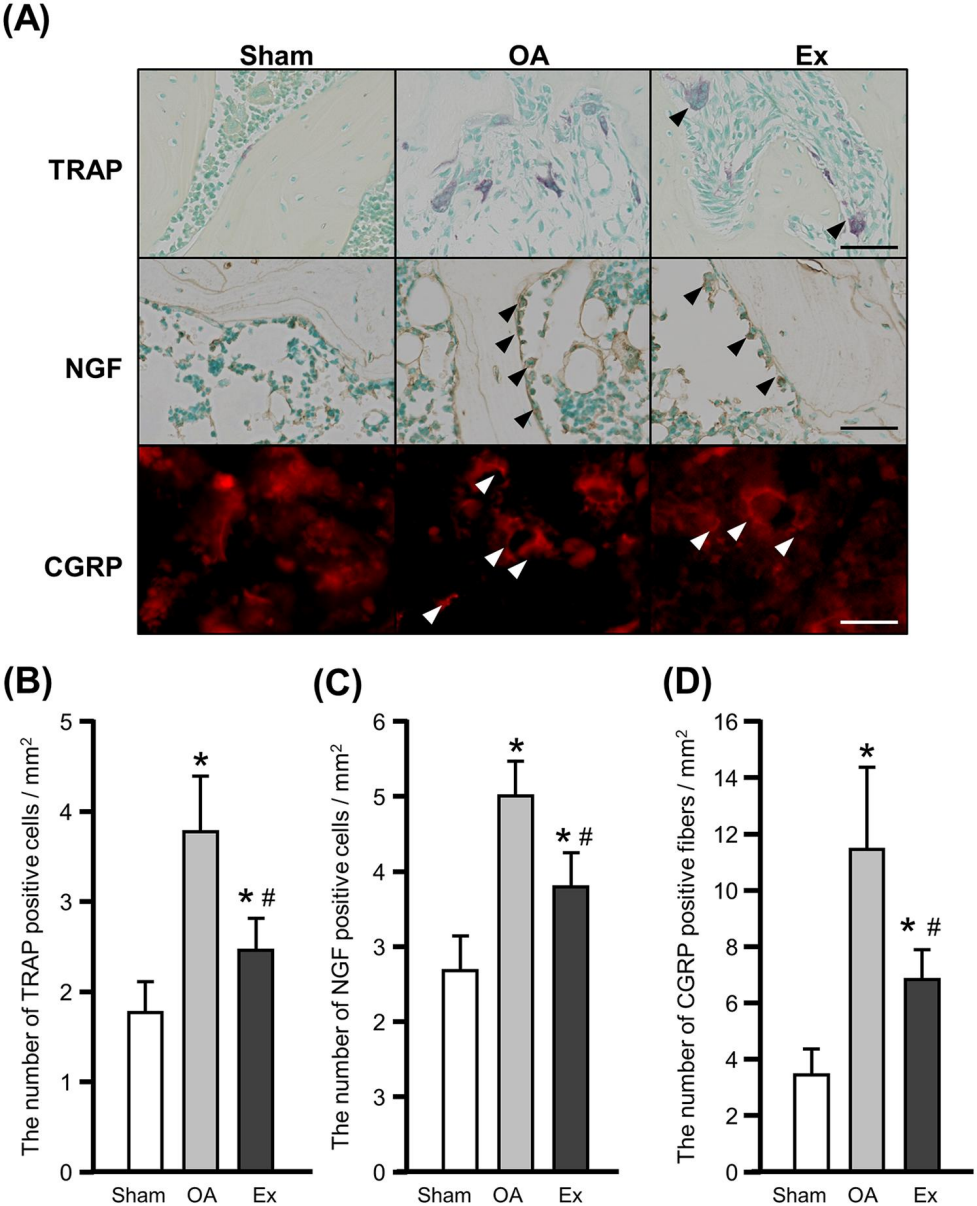
Histochemical, immunohistochemical, and immunofluorescence findings and quantification of TRAP- and NGF-positive cells and CGRP-positive fibers in the right knee joint at 56 days after the injection of MIA. **(A)** Representative images of tartrate-resistant acid phosphatase (TRAP) staining (upper), nerve growth factor (NGF) (middle), and calcitonin gene-related peptide (CGRP) (lower). *Arrowhead:* positive cells. Bar: 30 µm. **(B)** The number of TRAP-positive cells. **(C)** The number of NGF-positive cells. **(D)** The number of CGRP-positive fibers. *vs. the Sham group, #vs. the OA group, *p* < 0.05.

We observed multinucleated giant TRAP-positive cells at the margins of the marrow cavity of subchondral bone from the OA and Ex rats (Figure 5A upper panels), indicating an increase in osteoclasts. NGF immunoreactivity was mainly detected in cell-associated structures within the subchondral bone marrow cavity. Because the sections were cut at 5 µm thickness, continuous fiber-like structures were not readily visualized, and positive signals were therefore defined as fluorescence localized around cell nuclei. NGF-positive cells were also frequently observed at the margins of the marrow cavities in these groups, whereas only few were observed in the sham group (Figure 5 middle panels).

Quantitative assessment demonstrated that the mean number of osteoclasts was 1.8 ± 0.4 cells/mm^2^ in the Sham group, 3.8 ± 0.6 cells/mm^2^ in the OA group, and 2.5 ± 0.4 cells/mm^2^ in the Ex group. Both OA and Ex groups showed significantly higher values than the Sham group (Sham vs. OA, *p* < 0.001; Sham vs. Ex, *p* < 0.001), while the Ex group exhibited significantly fewer osteoclasts than the OA group (*p* < 0.01, Figure 5B). Similarly, the mean number of NGF-positive cells was 2.7 ± 0.5 cells/mm^2^ in Sham, 5.0 ± 0.5 cells/mm^2^ in OA, and 3.8 ± 0.4 cells/mm^2^ in Ex. The OA and Ex groups had significantly higher counts compared with Sham (*p* < 0.001 for both), whereas Ex had significantly lower values than OA (*p* < 0.001, Figure 5C).

The immunofluorescence staining for CGRP showed more positive fibers in the OA and Ex groups compared to the sham group (Figure 5A lower panels). The mean values were 3.4 ± 1.0 fiber/mm^2^ in Sham, 11.4 ± 3.1 fiber/mm^2^ in OA, and 6.9 ± 1.0 fiber/mm^2^ in Ex. Both OA and Ex groups had significantly increased CGRP-positive fibers relative to Sham (*p* < 0.001), and the Ex group again showed significantly reduced values compared with OA (*p* < 0.001, Figure 5D).

### pNR1 fluorescence immunostaining results

**Figure 6:**
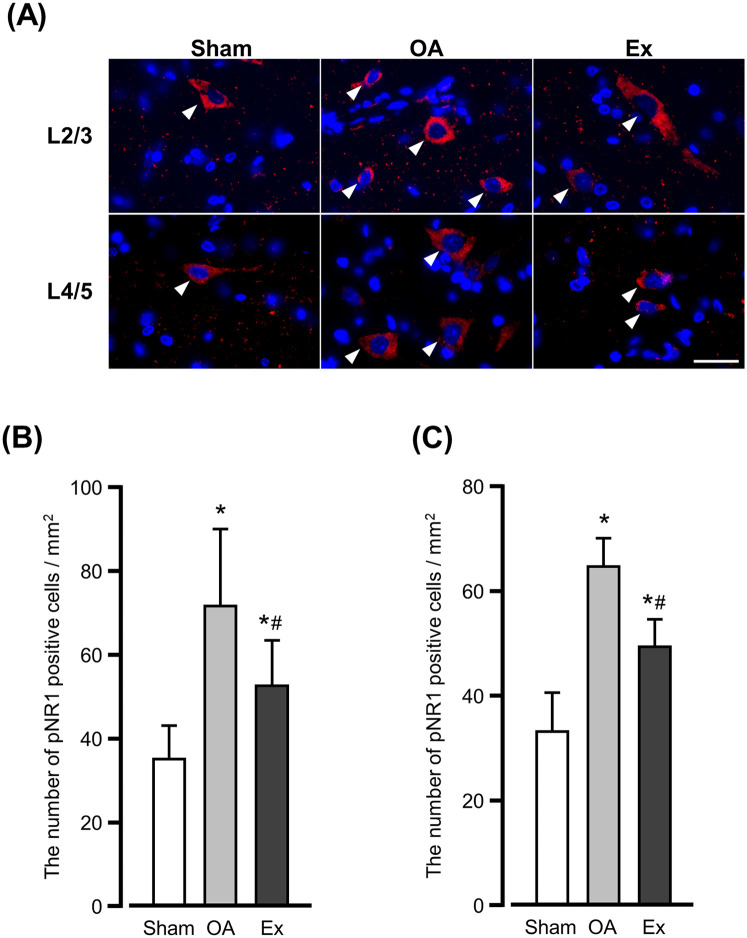
Immunofluorescence findings for phosphorylated NR1 (pNR1)-positive cells in the spinal dorsal horn. **(A)** Representative of immunofluorescence findings in the 2nd/3rd (upper) and 4th/5th (lower). *Arrowhead:* positive cells. Bar: 30 µm. The number of pNR1-positive cells in the spinal dorsal horn of L2/3 **(B)** and L4/5 **(C)** at 56 days after the MIA injection. *vs. the Sham group, #vs. the OA group, *p* < 0.05.

The fluorescence immunostaining for phosphorylated NR1 (pNR1) in the 2nd/3rd and 4th/5th lumbar spinal cords showed different distribution patterns in the OA and Ex groups. In the sham group, pNR1-positive cells were sparsely distributed throughout the dorsal horn of the spinal cord (Figure 6A left panels), whereas in the OA group a high density of positive cells was observed throughout the dorsal horn (Figure 6A center panels). The Ex group also showed positive cells throughout the dorsal horn, but their density was significantly lower compared to the OA group (Figure 6A right panels).

The quantitative analysis demonstrated that the number of pNR1-positive cells per mm^2^ in the 2nd/3rd lumbar dorsal horn was 35.3 ± 8.0 cells/mm^2^ in the Sham group, 72.0 ± 18.7 cells/mm^2^ in the OA group, and 52.8 ± 10.9 cells/mm^2^ in the Ex group. Both OA and Ex groups showed significantly higher values than Sham (*p* < 0.001 for each), whereas the Ex group had significantly fewer positive cells than the OA group (*p* < 0.001, Figure 6B).

A similar trend was observed in the 4th/5th lumbar dorsal horn: the values were 33.8 ± 7.3 cells/mm^2^ for Sham, 64.4 ± 5.7 cells/mm^2^ for OA, and 49.3 ± 5.4 cells/mm^2^ for Ex. Again, OA and Ex groups showed markedly higher counts compared to Sham (*p* < 0.001), and the Ex group demonstrated a significant reduction compared with OA (*p* < 0.001, Figure 6C).

## Discussion

We investigated the effects of low-intensity muscle contraction exercise on pain sensitivity, the peripheral pathology of knee OA pain, and central sensitization in the spinal dorsal horn by using a rat model of end-stage knee OA. Our findings indicate that (i) the low-intensity muscle contraction exercise contributed to the improvement of synovitis in the rats and abnormal changes in the subchondral bone and central sensitization levels, and (ii) low-intensity muscle contraction exercises may be an effective strategy for the management of knee OA pain.

The data of the PPT at the rat knee joint and the PWR at the plantar region suggest that low-intensity muscle contraction exercise is effective for improving the pain sensitivity of the knee joint and secondary hyperalgesia in the planta, even at the end-stage of knee OA. Generally, in models that use MIA, the PWR of the plantar region reflects central sensitization in the spinal cord and is a type of secondary hyperalgesia because the reflex is elicited from the uninjured region, i.e., the hindlimb, away from the injured knee (24, 25). The results of the present study suggest that low-intensity muscle contraction exercises not only improve pain sensitivity in the affected joint but also positively influence the central sensitization process. Our findings also highlight the potential of low-intensity exercise as a therapeutic approach to manage chronic pain and alleviate central sensitization in knee OA.

We conducted histological evaluations to determine whether the hypoalgesia of the low-intensity muscle contraction exercise was associated with changes in the degeneration of articular cartilage and subchondral bone, and the histological findings showed a similar pattern of degeneration of articular cartilage and subchondral bone in the OA and Ex groups. In other words, the low-intensity muscle contraction exercises at the end-stage of knee OA had no effect on the protection against or the progression of the articular cartilage and subchondral bone damage. Nevertheless, the low-intensity muscle contraction exercise effectively alleviated pain sensitivity in the end-stage knee OA rat model. These results suggest that the effects of low-intensity muscle contraction exercise may occur independently of structural changes in the joint, as shown by toluidine blue staining. This supports the potential of such exercise as a therapeutic approach for managing pain in humans with end-stage knee OA, regardless of the extent of joint degeneration.

Our immunohistochemical analysis of synovium macrophages demonstrated that the low-intensity muscle contraction exercise reduced the numbers of total and M1 macrophages while increasing M2 macrophages in the synovial membrane in rats with end-stage knee OA. Macrophage infiltration into the synovial membrane is a hallmark of knee OA (26), and synovitis is commonly observed in this condition. In particular, M1 macrophages produce proinflammatory cytokines [e.g., interleukin (IL)-1β] that amplify inflammatory responses and exacerbate synovitis (26). Conversely, M2 macrophages secrete anti-inflammatory cytokines such as IL-10 which suppress inflammation and are thought to mitigate synovitis (27). Clinical studies have shown that patients with end-stage knee OA and severe pain exhibit a significant increase in M1 macrophages and a notable decrease in M2 macrophages within the synovium compared with healthy individuals (28). Based on our present results, the observed changes in synovial macrophage numbers may partly contribute to the improvement in pain sensitivity following exercise. However, this interpretation should be made with caution, as cytokine assays and detailed immune profiling were not included in this study. Without such data, the involvement of specific macrophage phenotypes (e.g., M1/M2 polarization) remains speculative. It should also be noted that CD11c + and CD206 + cell counts in the Ex group appeared higher than CD68 + cell counts. This discrepancy may reflect section-to-section variability and marker-specific differences in antigen detectability. Therefore, our interpretation focuses on consistent between-group differences across markers, rather than on absolute numerical comparisons. Moreover, we recognize that using single-marker immunohistochemistry to infer macrophage polarization (M1 vs. M2) has inherent limitations. While CD11c and CD206 are commonly used in the field, their specificity is moderate and does not exclude other cell types or intermediate phenotypes. Ideally, double or triple labeling (e.g., CD68 with CD11c or CD206) would provide more definitive evidence of macrophage subtype identification. Future studies employing dual-labeling, cytokine measurements, and comprehensive immune profiling will be important to confirm the mechanisms by which macrophage modulation contributes to pain relief.

Similar to the findings regarding macrophages, the Ex group's numbers of osteoclasts, NGF-positive cells, and CGRP-positive fibers in the subchondral bone were significantly lower than those in the OA group. This may indicate that in addition to synovitis, osteoclast changes in the subchondral bone (although not confirmed by toluidine blue staining) contribute to the improvement of pain sensitivity. Indeed, a clinical study demonstrated that the severity of subchondral bone lesions observed via magnetic resonance imaging (MRI) was positively correlated with the pain intensity (29). One of the primary mechanisms underlying pain originating from the subchondral bone in knee OA involves the increased number and activation of osteoclasts and the upregulation of NGF (30). The administration of bisphosphonates in rat OA models has been shown to reduce the number of osteoclasts in the subchondral bone and improve the pain threshold (31). The osteoclasts release NGF in the subchondral bone as well as protons involved in pain generation (14). It has also been shown that rats with MIA-induced OA have an increased number of CGRP-immunoreactive neurons in the subchondral bone (32). In short, these findings suggest that (*i*) the increase and activation of osteoclasts in the subchondral bone leads to an increase in NGF and a subsequent increase in the number of CGRP-positive primary sensory neurons, and (*ii*) these changes are responsible for OA pain of subchondral origin. Low-intensity muscle contraction exercise then reduces these changes, which may also contribute to improved pain sensitivity.

The increased expression of pNR1 in the dorsal horn of the spinal cord is closely associated with hyperalgesia and serves as a reliable indicator of central sensitization (33, 34). The data regarding pNR1-positive cells in the present study suggest that the low-intensity muscle contraction exercise applied to rats with end-stage knee OA suppressed central sensitization in the spinal dorsal horn. Sustained and enhanced nociceptive stimulation drive central sensitization at the spinal level through the activation of NMDA receptor (35), and NMDA receptor antagonists have effectively alleviated pain-related behavior in animal models and clinical situations (36, 37). In the present study's OA group, the persistence of synovitis and associated inflammation likely caused sustained nociceptive input, contributing to the development of central sensitization in the spinal segments governing the knee joint. This central sensitization may have extended to spinal segments innervating the plantar area, reflecting the secondary hyperalgesia. In contrast, in the Ex group, the observed suppression of synovitis and pathological changes in the subchondral bone likely reduced the nociceptive input from peripheral tissues to secondary nociceptive neurons. This attenuation of nociceptive input could have led to a reduction in central sensitization at the spinal level. The pain relief and reduction in secondary hyperalgesia observed in the Ex group may be attributable to the suppression of central sensitization through the alleviation of peripheral pathological conditions.

Although the precise mechanisms by which low-intensity muscle contraction exercise induced the observed changes remain unclear, prior studies provide potential explanations. During contraction, skeletal muscles secrete anti-inflammatory cytokines such as IL-10 and IL-6 as well as anti-inflammatory hormones such as brain natriuretic peptide and irisin (38). These substances have been reported to promote the conversion of pro-inflammatory M1 macrophages to anti-inflammatory M2 macrophages, particularly in adipose tissue (39). It has been hypothesized that these skeletal muscle-derived factors circulate through the bloodstream to exert systemic anti-inflammatory effects on other tissues and organs (40). In this context, it is plausible that the anti-inflammatory cytokines and hormones released during muscle contraction may play a significant role in modulating the behavior of macrophages and osteoclasts. This process could contribute to the alleviation of synovitis and pain; however, this potential mechanism was not explored in this study and further studies are needed to directly test this hypothesis.

Our study has some limitations to address. The first limitation is the lack of detailed information on the dynamics of pain-related molecules. During synovitis, the increased number of M1 macrophages drives the expression of pro-inflammatory cytokines such as IL-1β, tumor necrosis factor (TNF)-α, and NGF, which collectively contribute to pain sensitization (41). Although our findings suggest that low-intensity muscle contraction exercise alleviates pain in the affected area by reducing the number of M1 macrophages, the changes in the levels and the roles of these specific pain-related molecules remain to be clarified.

Second, this study is the exclusive use of male rats. Osteoarthritis is more prevalent and often more severe in women compared to men (42). Moreover, in the monosodium iodoacetate (MIA)-induced OA pain model, aged female rats exhibit more profound and long-lasting hyperalgesia than males (43). Therefore, the lack of inclusion of female animals limits the generalizability of our findings, and future studies should include both sexes to elucidate sex-specific differences in pain responses and intervention efficacy.

Another limitation is that NGF and CGRP were evaluated by immunohistochemistry, which primarily reflects the presence of positive cells rather than the actual levels of secreted proteins. Direct quantification of NGF and CGRP (e.g., by ELISA or immunoblotting) would provide stronger evidence regarding their contribution to local hyperalgesia. Future studies will therefore be needed to directly assess secreted NGF and CGRP levels.

## Conclusion

The results of this study demonstrated that low-intensity muscle contraction exercise has the potential to alleviate pain and improve pathological conditions in a rat model of end-stage knee osteoarthritis (OA). The exercise intervention significantly improved pain thresholds in both the affected knee joint and the plantar region, suggesting its effectiveness in mitigating both peripheral and central sensitization. Our findings collectively indicate that low-intensity muscle contraction exercise ameliorates pain through a multifaceted mechanism involving improvements in peripheral pathology and a suppression of central sensitization. While these results provide valuable insights into the potential therapeutic effects of exercise for end-stage knee OA, further studies are necessary to clarify the dynamics of pain-related molecules and cytokines as well as the impact of various exercise conditions on pain-reduction mechanisms.

## Data Availability

The raw data supporting the conclusions of this article will be made available by the authors, without undue reservation.
