# Supplementary material for: Low-intensity muscle contraction exercise reduces pain sensitivity by modulating peripheral pathology and spinal sensitization in end-stage knee osteoarthritis rats
Source: Front Pain Res (Lausanne). 2025 Sep 29;6:1644177. doi: 10.3389/fpain.2025.1644177 (PMC12515908; doi:10.3389/fpain.2025.1644177)
Supplement: Supplementary file 1 [file Table1.docx]

|  | PPT | | | | | |  | PWR | | | | | |
| --- | --- | --- | --- | --- | --- | --- | --- | --- | --- | --- | --- | --- | --- |
| Day | Sham | OA | Ex | Sham  vs. OA | Sham  vs. Ex | OA  vs. Ex |  | Sham | OA | Ex | Sham  vs. OA | Sham  vs. Ex | OA  vs. Ex |
| B.L. | 206.7 ± 4.3 | 206.9 ± 4.7 | 207.5 ± 4.0 | 1.000 | 1.000 | 1.000 |  | 59.3 ± 2.3 | 59.1 ± 2.1 | 59.1 ± 2.4 | 1.000 | 1.000 | 1.000 |
| D04 | 207.0 ± 3.8 | 117.9 ± 6.1 | 118.9 ± 6.4 | 0.000 | 0.000 | 1.000 |  | 60.2 ± 1.5 | 20.2 ± 2.2 | 20.5 ± 1.8 | 0.000 | 0.000 | 1.000 |
| D07 | 208.7 ± 2.3 | 121.1 ± 5.6 | 116.1 ± 7.8 | 0.000 | 0.000 | 0.190 |  | 59.1 ± 2.4 | 19.9 ± 2.4 | 19.9 ± 1.3 | 0.000 | 0.000 | 1.000 |
| D14 | 205.7 ± 3.5 | 119.1 ± 5.7 | 122.0 ± 6.5 | 0.000 | 0.000 | 0.697 |  | 59.6 ± 1.5 | 22.0 ± 2.3 | 22.3 ± 1.0 | 0.000 | 0.000 | 1.000 |
| D21 | 205.1 ± 5.4 | 117.7 ± 4.5 | 120.4 ± 5.8 | 0.000 | 0.000 | 0.671 |  | 61.0 ± 0.8 | 22.8 ± 1.9 | 22.9 ± 2.4 | 0.000 | 0.000 | 1.000 |
| D28 | 207.4 ± 3.5 | 119.7 ± 5.8 | 122.9 ± 4.4 | 0.000 | 0.000 | 0.406 |  | 61.0 ± 1.1 | 23.1 ± 1.1 | 22.9 ± 2.0 | 0.000 | 0.000 | 1.000 |
| D35 | 204.4 ± 4.3 | 120.9 ± 6.9 | 133.4 ± 3.7 | 0.000 | 0.000 | 0.000 |  | 61.1 ± 1.3 | 21.8 ± 2.1 | 24.0 ± 1.3 | 0.000 | 0.000 | 0.012 |
| D42 | 203.3 ± 5.4 | 120.5 ± 5.0 | 146.7 ± 6.4 | 0.000 | 0.000 | 0.000 |  | 60.9 ± 1.1 | 22.9 ± 1.3 | 29.6 ± 2.1 | 0.000 | 0.000 | 0.000 |
| D49 | 203.5 ± 2.6 | 120.4 ± 4.6 | 152.5 ± 5.0 | 0.000 | 0.000 | 0.000 |  | 59.8 ± 1.3 | 23.5 ± 1.0 | 33.4 ± 2.1 | 0.000 | 0.000 | 0.000 |
| D56 | 205.2 ± 4.5 | 121.3 ± 5.9 | 158.3 ± 8.5 | 0.000 | 0.000 | 0.000 |  | 60.3 ± 1.5 | 23.1 ± 1.5 | 36.9 ± 2.6 | 0.000 | 0.000 | 0.000 |

Supplementary Table 1. Pain behavioral outcomes (PPT and PWR) across groups and timepoints

All data are expressed in grams (g). The columns “Sham vs. OA,” “Sham vs. Ex,” and “OA vs. Ex” indicate p values for between-group comparisons. Statistical analyses were performed using repeated-measures ANOVA followed by Bonferroni post hoc test.
