# Supplementary material for: Low-intensity muscle contraction exercise reduces pain sensitivity by modulating peripheral pathology and spinal sensitization in end-stage knee osteoarthritis rats
Source: Front Pain Res (Lausanne). 2025 Sep 29;6:1644177. doi: 10.3389/fpain.2025.1644177 (PMC12515908; doi:10.3389/fpain.2025.1644177)
Supplement: Supplementary file 2 [file Table2.docx]

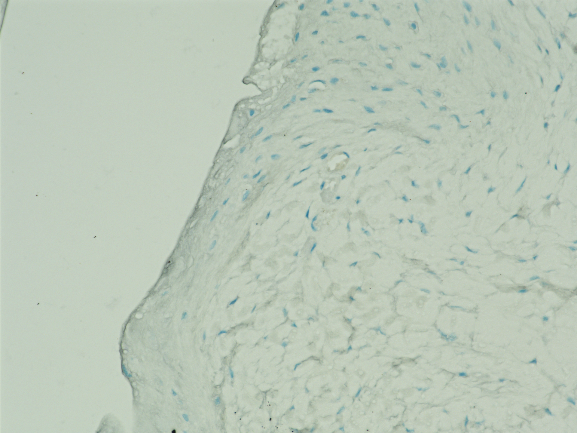

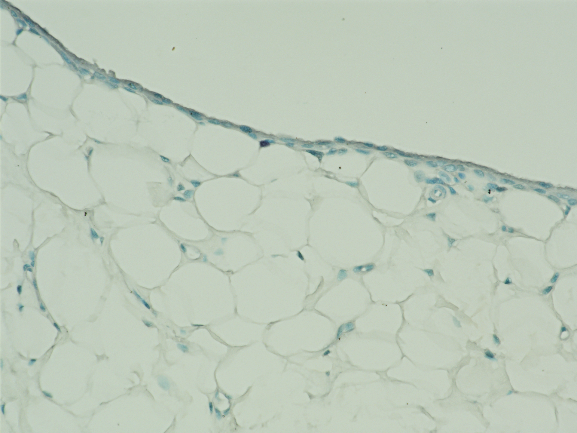


**B**

**A**


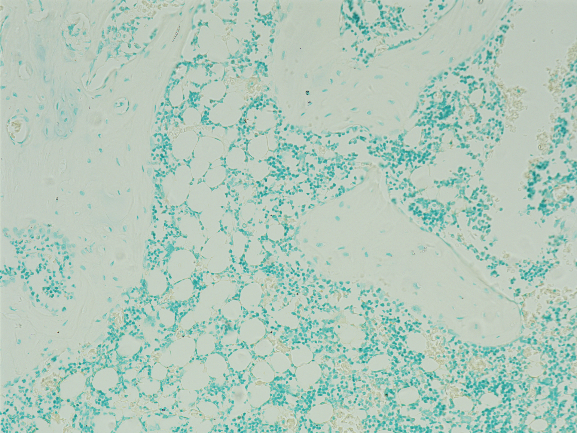

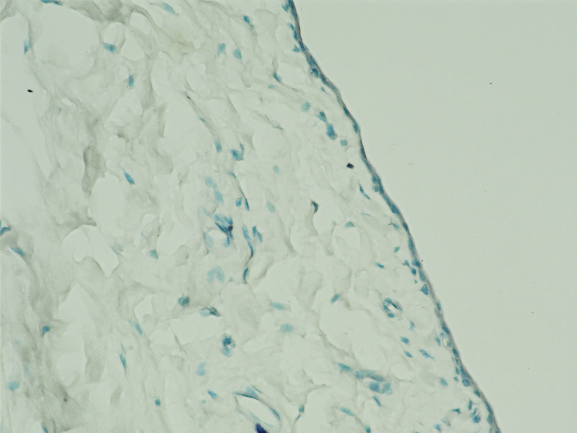


**D**

**C**

Supplemental Figure 1. Representative negative control images.

Representative sections of synovium and subchondral bone stained with control IgG instead of primary antibodies for CD68 (A), CD11c (B), CD206 (C), and NGF (D). No specific staining was observed, confirming the specificity of the immunohistochemical signals presented in the main figures. Scale bar = 10 μm (A, B, and C), 50 μm (D)
